# Neuromuscular blocking agents for acute respiratory distress syndrome: an updated meta-analysis of randomized controlled trials

**DOI:** 10.1186/s12931-020-1287-4

**Published:** 2020-01-13

**Authors:** Zhongjun Zheng, Libing Jiang, Song Zhang, Christophe Guervilly, Mao Zhang, Xia Feng, Jianbo Ding

**Affiliations:** 1 Department of Emergency Medicine, The Second Affiliated Hospital, Zhejiang University School of Medicine, Jiefang road 88, Hangzhou, 310009 China; 2 Department of Intensive Care Unit, Hospital of Chengdu University of Traditional Chinese Medicine, Chengdu, 610072 China; 3 Medical Intensive Care Unit, North Hospital, APHM, Marseille, France; 4 CEReSS, Center for Studies and Research on Health Services and Quality of Life EA3279, Aix-Marseille University, Marseille, France; 5 Department of Respiratory, The Third People’s Hospital of Hangzhou, Hangzhou, 310009 China

**Keywords:** Neuromuscular blocking agents (NMBAs), Acute respiratory distress syndrome (ARDS), Mortality, Meta-analysis

## Abstract

**Backgrounds:**

The aim of this study is investigating the benefits and harms of neuromuscular blocking agents (NMBAs) in patients with acute respiratory distress syndrome (ARDS).

**Methods:**

We comprehensively searched PubMed, EMBASE, and Cochrane library for randomized controlled trials comparing NMBAs to any other comparator. We pooled data using relative risk (RR) for dichotomous outcomes and weighted mean difference (WMD) for continuous outcomes, with 95% confidence intervals. We assessed the quality of included studies using the Cochrane tool and levels of evidence using the GRADE method.

**Results:**

Finally, six RCTs (*n* = 1557 patients) were eligible for analysis. The results showed NMBAs use was not associated with reduced 28 days mortality (RR 0.78; 95% CI, 0.58 to 1.06; *P* = 0.11), 90 days mortality (RR, 0.92; 95% CI, 0.81 to 1.04; *P* = 0.16), and intensive care unit (ICU) mortality (RR, 0.90; 95% CI, 0.79 to 1.03; *P* = 0.13) in patients with ARDS. However, 21–28 days mortality was slightly lower in patients received NMBAs (RR 0.73; 95% CI, 0.54 to 0.99; *P* = 0.04; I^2^ = 53%). Besides, NMBAs use could improve the PaO_2_/FiO_2_ ratio at 48 and 72 h, decrease plateau pressure and PEEP at 72 h. Additionally, NMBAs had no significant effects on days free of ventilation at day 28 (WMD, 0.55; 95% CI, − 0.46 to 1.57; *P* = 0.29), days not in ICU at day 28 (WMD, 0.12; 95% CI, − 0.85 to 1.08; *P* = 0.82), ICU-acquired weakness (RR, 1.23; 95% CI, 0.99 to 1.93; *P* = 0.06). Finally, NMBAs use was associated with a lower risk of barotrauma (RR, 0.55; 95% CI, 0.35 to 0.85; *P* = 0.007).

**Conclusion:**

In patients with respiratory distress syndrome, NMBAs may be beneficial in reverse refractory hypoxemia and may be associated with reduced short-term mortality and incidence of barotrauma. However, there is no significant effects of NMBAs on mid-term and long-term mortality, and further studies are required.

## Backgrounds

Acute respiratory distress syndrome (ARDS) is a life-threatening condition characterized by refractory acute hypoxemia [1]. It is a major cause of morbidity and mortality in intensive care unit (ICU) [2–4]. A number of interventions have been proposed in the past decade; however, few of them obtained strong recommendation [5, 6]. Only lung-protective mechanical ventilation strategy has been proven beneficial for prognosis of these patients [5, 7]. Neuromuscular blocking agents (NMBAs) may be a useful therapeutic strategy in patients with ARDS [8]. The ARDS et Curarisation Systematique (ACURASYS) trial conducted in 2010 found early administration of a 48-h infusion of NMBA was associated with a lower risk of death in patients with moderate-to-severe ARDS [9]. It is important to realize that patients in the control group in this study received deep sedation, and this is inconsistent with the current guidelines [10, 11]. A meta-analysis including 5 studies systematically reviewed the effects of NMBAs on ARDS. They concluded the application of NMBAs could reduce the mortality of patients with moderate-to-severe ARDS [12]. However, the results of this meta-analysis are mainly affected by the ACURASYS trial [9]. Based on the limited evidence and potential adverse events, NMBAs is only weakly recommended in the current guidelines [13–15]. A new multi-center randomized control study (Reevaluation of Systemic Early Neuromuscular Blockade [ROSE] trial) just published recently [16]. Thus, the main aim of this study is to investigate the effects of NMBAs in moderate-to-severe ARDS by an update meta-analysis.

## Methods

This study was performed and reported according to the Preferred Reporting Items for Systematic Reviews and Meta-Analyses (PRISMA) guidelines (An additional file shows the detailed information on PRISMA checklist [see Additional file 1: Figure S1]). The review protocol was registered in the International Prospective Register of Systematic Reviews (PROSPERO, CRD42019137195).

### Literature search strategies

PubMed, EMBASE and Cochrane library were searched from their inception to Jun 2019. There was no language limitation. Additional file 1: Table S1 shows the detailed literature search strategies. The reference lists of related articles were searched for additional studies. In addition, we searched Clinical.gov for ongoing studies and unpublished data. Two authors (ZJZ, LBJ) independently performed literature search, any disagreement was resolved by discussion or consultation with a third author (MZ).

### Study selection and data extraction

Two authors (ZJZ, LBJ) did study selection and data extraction. And disagreement was resolved by discussion or consultation with another author (SZ). Firstly, we excluded duplicated articles. Then, we excluded clearly non-relevant articles by screening titles and abstracts. Finally, we included eligible studies by reading the full-text of remaining studies. The following data were extracted: name of first author, publication year, country, sample size, characteristics of included patients, intervention strategies, control strategies, endpoints and other items necessary for quality evaluation. If necessary, we would contact the author of original articles for additional data.

### Inclusion criteria

**Patients:** adult acute respiratory distress syndrome defined by each study.

**Intervention:** neuromuscular blocking agents regardless of drug type, dose, or use duration.

**Control:** none or placebo.

**Endpoints:** the primary endpoints included 21 to 28 days mortality (short-term mortality), ICU mortality (mid-term mortality) and 90 days mortality (long-term mortality). The secondary endpoints included respiratory parameter such as PaO_2_/FIO_2_, plateau pressure (Pplat), and positive end-expiratory pressure (PEEP) at 24 h, 48 h, 72 h; days free of ventilation at day 28 (DFV); days not in ICU at day 28; incidence of biotrauma and ICU-acquired weakness.

### Study quality evaluation

Two authors (SZ and XF) evaluated the qualities of all eligible studies using the Cochrane Risk of Bias Tool. The following domains were evaluated: random sequence generation, allocation concealment, blinding of participants and personnel, blinding of outcome assessment, incomplete outcome data, selective reporting, and other bias. Each domain was classified as low risk of bias, unclear risk of bias and high risk of bias.

### Statistical analysis

Relative risks (RRs) and weighted mean differences (WMDs) with 95% confidence intervals (CIs) were used to estimate the pooled effect of dichotomous variables and continuous variables respectively. Heterogeneity between studies was assessed using the Q statistic and I^2^ statistic. *P* < 0.10 or I^2^ ≥ 50% indicated there was significant heterogeneity between studies and random effect model was used, otherwise, the fixed effect model would be used. If there was no significant heterogeneity, we would perform additional sensitivity analyses using random effects models to test the robustness of the results. A different number of studies were included in the various primary and secondary end-points analysis, detailed citations for included studies were shown in the different results. Publication bias would be assessed using Funnel plot and Egger test, if the number of included studies was over 10.

In order to minimize the risks of random errors resulting from sparse data during repetitive test, we performed trial sequence analysis (TSA) and calculated the optimal information size for the primary endpoints. In addition, we constructed the adjusted boundary line for favoring the NMBAs or controls to decide whether the meta-analysis could be terminated early. The optimal information size was calculated using α = 0.05 (two-sided), β = 0.20 (power 80%), the anticipated relative risk reduction, and the incidence in control arm.

A *p* value less than 0.05 was considered statistically significant. All analyses were performed in RevMan 5.3 (Cochrane Collaboration, Oxford) software. TSA was perfomed using Trial Sequential Analysis v.0.9.5.10 beta (Copenhagen Trial Unit, Centre for Clinical Intervention Research, Rigshospitalet, Copenhagen, Denmark, available from www.ctu.dk/tsa).

### Grade

GRADE (Grades of Recommendation, Assessment, Development, and Evaluation) was used to evaluate the level of evidence. All patient center endpoints (21–28 days mortality, ICU mortality and 90 days mortality, DFV at day 28, days not in ICU at day 28; incidence of biotrauma and ICU-acquired weakness) were graded as high, moderate, low, and very low. This process was performed on GRADEpro GDT (https://gradepro.org/).

## Results

**Fig. 1:**
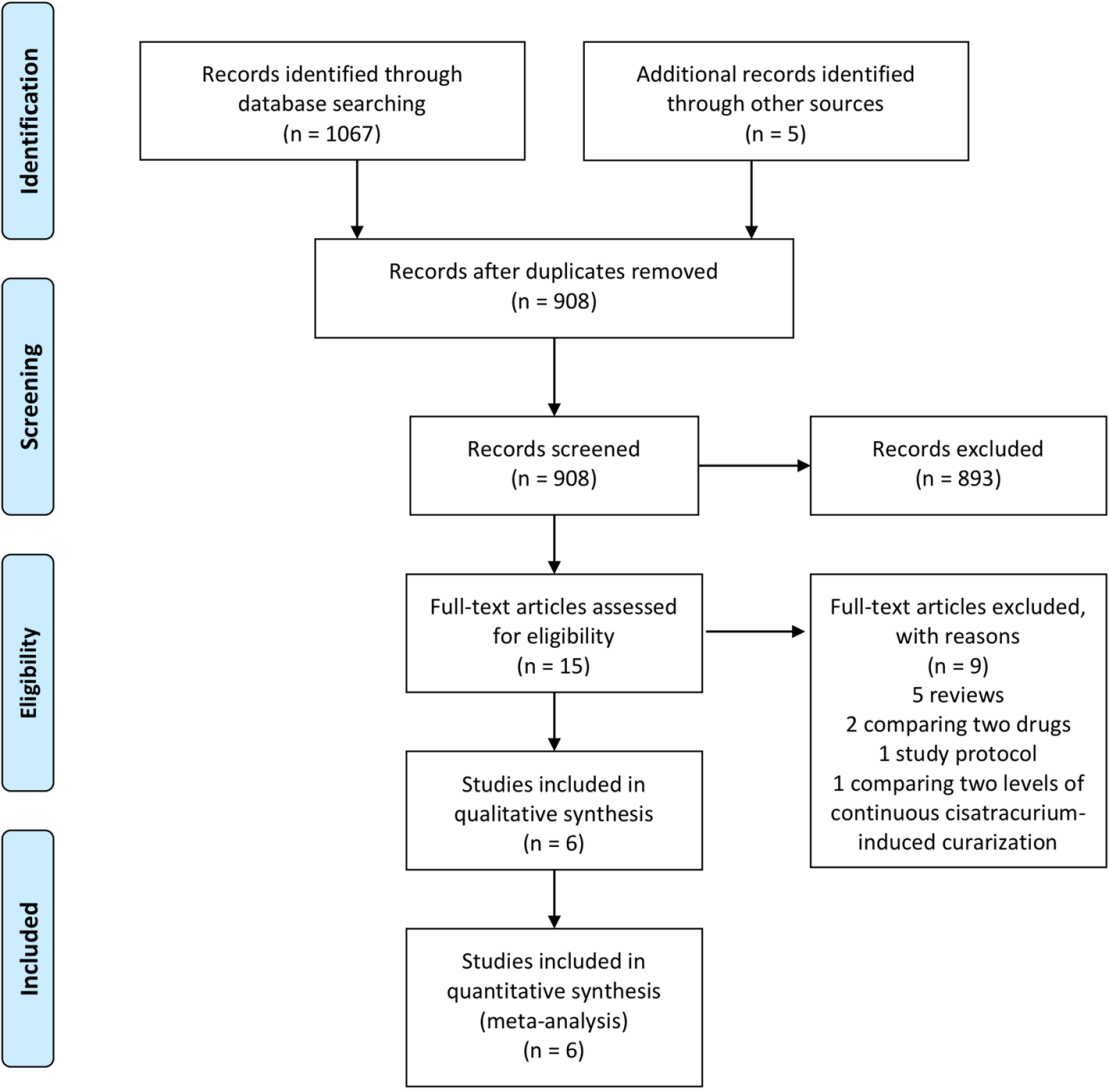
PRISMA flowchart for the systematic review and meta-analysis

A total of six studies containing 1557 patients were included in the analysis [9, 16–20]. Figure 1 shows the detailed information of literature selection. All the data were obtained from published papers, including a meta-analysis [21] or by contacting the author of original articles. Four studies [9, 17–19] performed in France, one study [20] performed in China, and one study [16] performed in United States of America. Vecuronium was used in one study [20], and cisatracurium was used in the remaining studies [9, 16–19]. Table 1, Additional file 1: Tables S2, S3 and S4 show the detailed characteristics of included studies.

**Table 1:** Characteristics of included studies

Study	Country	Patients (N)	NMBAs	Experimental intervention	Control intervention	Ventilation Strategy	Definition of ARDS
Gainnier 2004 [17]	France	56	Cisatracurium	A bolus of 50 mg followed by 5 μg∙kg^− 1^∙ min^− 1^ infusion for 48 h.	An infusion of saline at a rate of 4 mL/h	Volume-assist/control (6–8 mL/kg)	American-European consensus definition (PaO_2_:FIO_2_ ratio < 150, PEEP ≥5 cm H_2_O)
Forel 2006 [18]	France	36	Cisatracurium	A bolus of 0.2 mg/kg followed by 5 μg∙kg^− 1^∙ min^− 1^ infusion for 48 h.	An infusion of saline at a rate of 4 mL/h	Volume-assist/control (4–8 mL/kg), plateau pressure of < 30 cm H_2_O	American-European consensus definition (PaO_2_:FIO_2_ ratio < 200, PEEP ≥5 cm H_2_O)
Papazian 2010 [9]	France	339	Cisatracurium	A bolus of 15 mg followed by 37.5 mg per hour for 48 h.	A bolus of 15 mg placebo followed 37.5 mg per hour for 48 h.	Volume-assist/control (6–8 mL/kg), plateau pressure of < 32 cm H_2_O	American-European consensus definition (PaO_2_:FIO_2_ ratio < 150, PEEP ≥5 cm H_2_O)
Lyu 2014 [20]	China	96	Vecuronium	A bolus of 0.1 mg∙kg − 1 followed by 0.05 mg∙kg^− 1^∙h^− 1^ for 24~ 48 h.	Usual treatment	Volume-assist/control (4–8 mL/kg), plateau pressure of < 30~35 cm H_2_O	The Berlin definition (PaO_2_: FIO_2_ ratio < 200, PEEP ≥5 cm H_2_O)
Guervilly 2017 [19]	France	24	Cisatracurium	A bolus of 15 mg followed by 37.5 mg per hour for 48 h	Usual treatment	Volume-assist/control (6 mL/kg)	The Berlin definition (PaO_2_: FIO_2_ ratio < 150, PEEP ≥5 cm H_2_O)
ROSE 2019 [16]	USA	1006	Cisatracurium	A bolus of 15 mg followed by 37.5 mg per hour for 48 h	Usual treatment	Volume-assist/control (6 mL/kg)	(PaO_2_:FIO_2_ ratio < 150, PEEP ≥8 cm H_2_O)

*N* number, *NMBA* Neuromuscular blocking agents, *ARDS* acute respiratory distress syndrome, *PEEP* positive end-expiratory pressure, *FIO*_*2*_ action of inspiration O_2_, *PaO*_*2*_ partial pressure of oxygen, *mg* milligram, *μg* microgram, *Kg* kilogram, *mL* milliliter, *cm* centimeter, *h* hour, *min* minute

### Risk of bias

Risk of bias of all included trials were assessed according to the Cochrane Collaboration tool. Most studies were judged at high risk of bias or unclear risk of bias in the domain of blinding. Detailed information about risk of bias of included studies are presented in the Additional file 1: Figures S2, S3, and Table S5.

### Publication bias

As only six studies were included in this meta-analysis, we did not evaluate the publication bias [22].

#### The primary endpoint

##### Effect of NMBAs on mortality

**21–28 days mortality.**

**Fig. 2:**
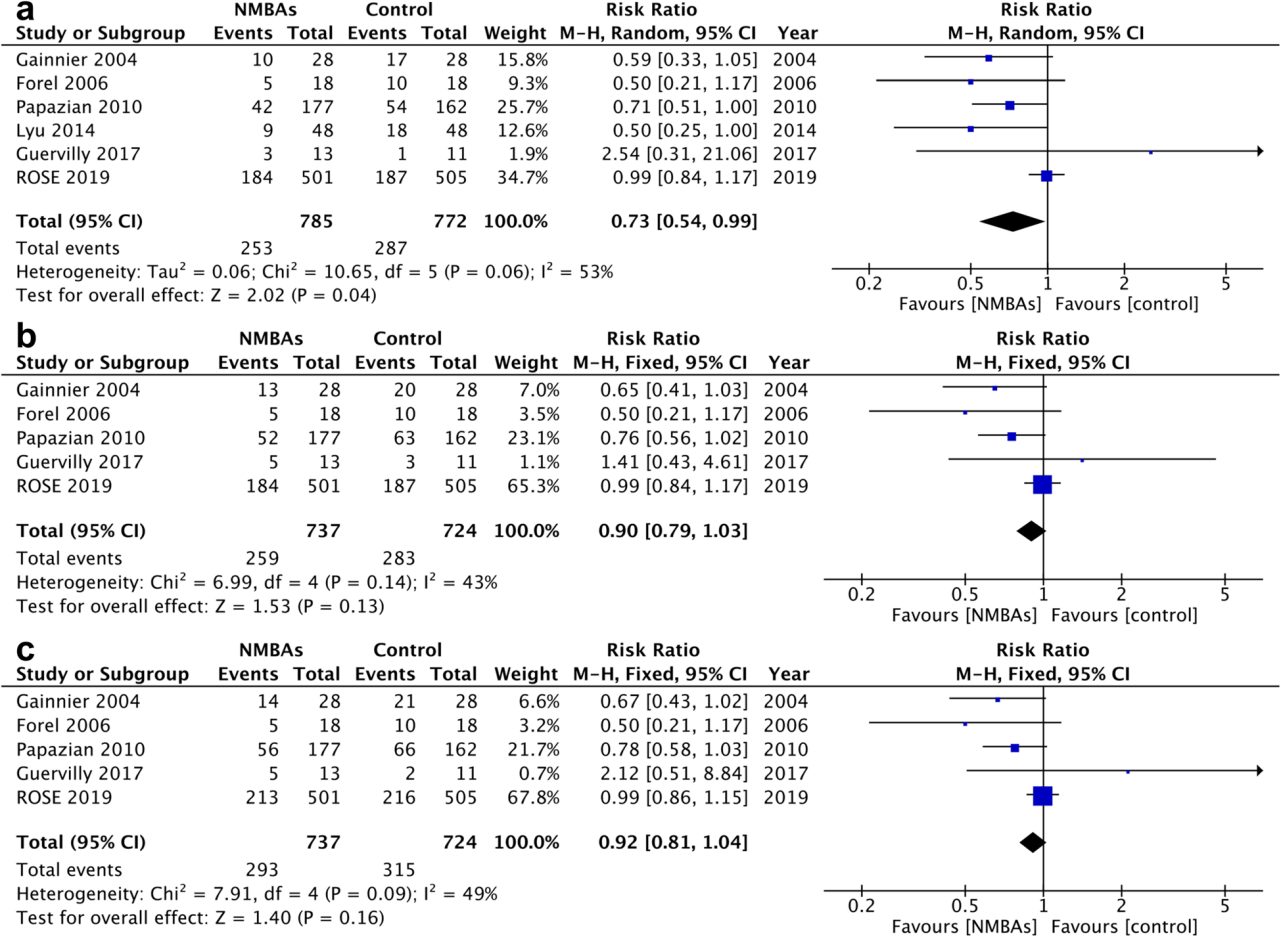
Forest plot for the mortality. (**a**, 21–28 days morality, random effect model; **b**, ICU mortality, fixed effect model; **c**, 90-day mortality, fixed effect model)

Six studies [9, 16–20] were eligible for 21–28 days mortality. Mortality at 21-day was reported only in one study [20], which was at high risk of bias. There was significant heterogeneity between studies and random-effect model was used. The rate of 21–28 days mortality was slightly lower in patients received NMBAs with moderate significant heterogeneity (RR 0.73; 95% CI, 0.54 to 0.99; *P* = 0.04; I^2^ = 53%; Fig. 2a). But there was no statistically significant effects of NMBAs on 28 days mortality, by excluding the trial which reported mortality at day 21 (RR, 0.78; 95% CI, 0.58 to 1.06; *P* = 0.11; I^2^ = 50%; Additional file 1: Figure S4).

**ICU mortality.**

Five studies [9, 16–19] were eligible for ICU mortality. There was no significant heterogeneity between studies for ICU mortality, and fixed effect model was used. There was no significant effects of NMBAs on ICU mortality (RR, 0.90; 95% CI, 0.79 to 1.03; *p* = 0.13; I^2^ = 43%; Fig. 2b). Sensitivity analysis showed similar results by performed with random effect model (Table 2).

**Table 2:** Summary of sensitivity analysis

Outcome	Number of trials (patients)	Number of events in each group (%)	Statistical method	Pooled effect estimates
21~28d mortality [9, 16–20]	6 (1557)	Intervention: 253/785 (32.2%) Control: 287/772 (37.2%)	M-H, Fixed, RR	0.87 (95% CI, 0.76 to 1.00); P = 0.05; I^2^ = 53%
28d mortality [9, 16–19]	5 (1461)	Intervention: 244/737 (33.1%) Control: 269/724 (37.2%)	M-H, Random, RR	0.78 (95% CI, 0.58 to 1.06); *P* = 0.11; I^2^ = 50%
28d mortality [9, 16–19]	5 (1461)	Intervention: 244/737 (33.1%) Control: 269/724 (37.2%)	M-H, Fixed, RR	0.90 (95% CI, 0.78 to 1.03); *P* = 0.12; I^2^ = 50%
ICU mortality [9, 16–19]	5 (1461)	Intervention: 259/737 (35.1%) Control: 283/724 (39.1%)	M-H, Random, RR	0.82 (95% CI, 0.65 to 1.04); *P* = 0.11; I^2^ = 43%
90d mortality [9, 16–19]	5 (1461)	Intervention: 293/737 (39.8%) Control: 315/724 (43.5%)	M-H, Random, RR	0.83 (95% CI, 0.65 to 1.06); *P* = 0.14; I^2^ = 49%
PaO_2_/FIO_2_ at 48 h^a^ [16–20]	5 (1170)	NA	IV, Random, WMD	17.71 (95% CI, − 0.74 to 36.15); *P* = 0.06; I^2^ = 67%
PaO_2_/FIO_2_ at 48 h^b^ [16–20]	5 (1170)	NA	IV, Random, WMD	26.98 (95% CI, 7.60 to 46.36); *P* = 0.006; I^2^ = 69%
PaO_2_/FIO_2_ at 48 h^c^ [16–20]	5 (1218)	NA	IV, Random, WMD	19.69 (95% CI, 3.61 to 35.78); *P* = 0.02; I^2^ = 70%
PaO_2_/FIO_2_ at 72 h [9, 16–18]	4 (1437)	NA	IV, Random, WMD	14.59 (95% CI, 2.40 to 26.78); P = 0.02; I^2^ = 37%
Pplat at 48 h [16–19]	4 (1122)	NA	IV, Random, WMD	−0.08 (95% CI, − 0.76 to 0.59); *P* = 0.81; I^2^ = 0%
Pplat at 72 h [9, 16–18]	4 (1437)	NA	IV, Random, WMD	−0.70 (95% CI, − 1.48 to 0.09); *P* = 0.08; I^2^ = 25%
PEEP at 48 h [16–19]	4 (1122)	NA	IV, Random, WMD	−0.39 (95% CI, − 0.87 to 0.09); P = 0.11; I^2^ = 0%
PEEP at 72 h [9, 16–18]	4 (1437)	NA	IV, Random, WMD	−0.43 (95% CI, − 0.83 to 0.03); *P* = 0.03; I^2^ = 0%
DFV at day 28 [9, 16–19]	5 (1461)	NA	IV, Random, WMD	0.70 (95% CI, − 0.51 to 1.92); *P* = 0.26; I^2^ = 13%
Days not in ICU at day 28 [9, 16, 19]	3 (1369)	NA	IV, Random, WMD	0.18 (95% CI, − 0.96 to 1.31); *P* = 0.76; I^2^ = 13%
Barotrauma [9, 16–19]	5 (1461)	Intervention: 29/737 (3.9%) Control: 52/724 (7.2%)	M-H, Random, RR	0.55 (95% CI, 0.35 to 0.85); *P* = 0.008; I^2^ = 0%
ICU-acquired weakness [9, 16–18]	4 (1437)	Intervention: 148/724 (20.4%) Control: 118/713 (16.5%)	M-H, Random, RR	1.23 (95% CI, 0.99 to 1.53); P = 0.06; I^2^ = 0%

*M-H* Mantel-Haenszel, *RR* Risk Ratio, *IV* Inverse Variance, *WMD* Weighted Mean Difference, *d* day, *CI* Confidence interval, *FIO*_*2*_ Action of inspiration O_2_, *PaO*_*2*_ Partial pressure of oxygen, *Pplat* Plateau pressure, *PEEP* Positive end-expiratory pressure, *DFV* Days free of ventilation, *ICU* Intensive care unit, *NA* Not available

^a^Included moderate ARDS of Lyu only

^b^Included severe ARDS of Lyu only

^c^Included both moderate and severe ARDS of Lyu

**90 days mortality.**

Five studies [9, 16–19] were eligible for 90 days mortality. There was no significant heterogeneity between studies for 90 days mortality, and fixed effect model was used. NMBAs use could not significantly reduce the 90 days mortality (RR, 0.92; 95% CI, 0.81 to 1.04; *P* = 0.16; I^2^ = 49%; Fig. 2c). Sensitivity analysis performed with random effect model showed similar results (Table 2).

The TSA showed the cumulative Z-curve neither crossed the monitoring boundary curve and nor reached the required information size, indicating further studies are required. Detailed information about TSA are presented in Additional file 1: Figures S5, S6 and S7.

#### The secondary endpoints

##### PaO_2_/FiO_2_

**Fig. 3:**
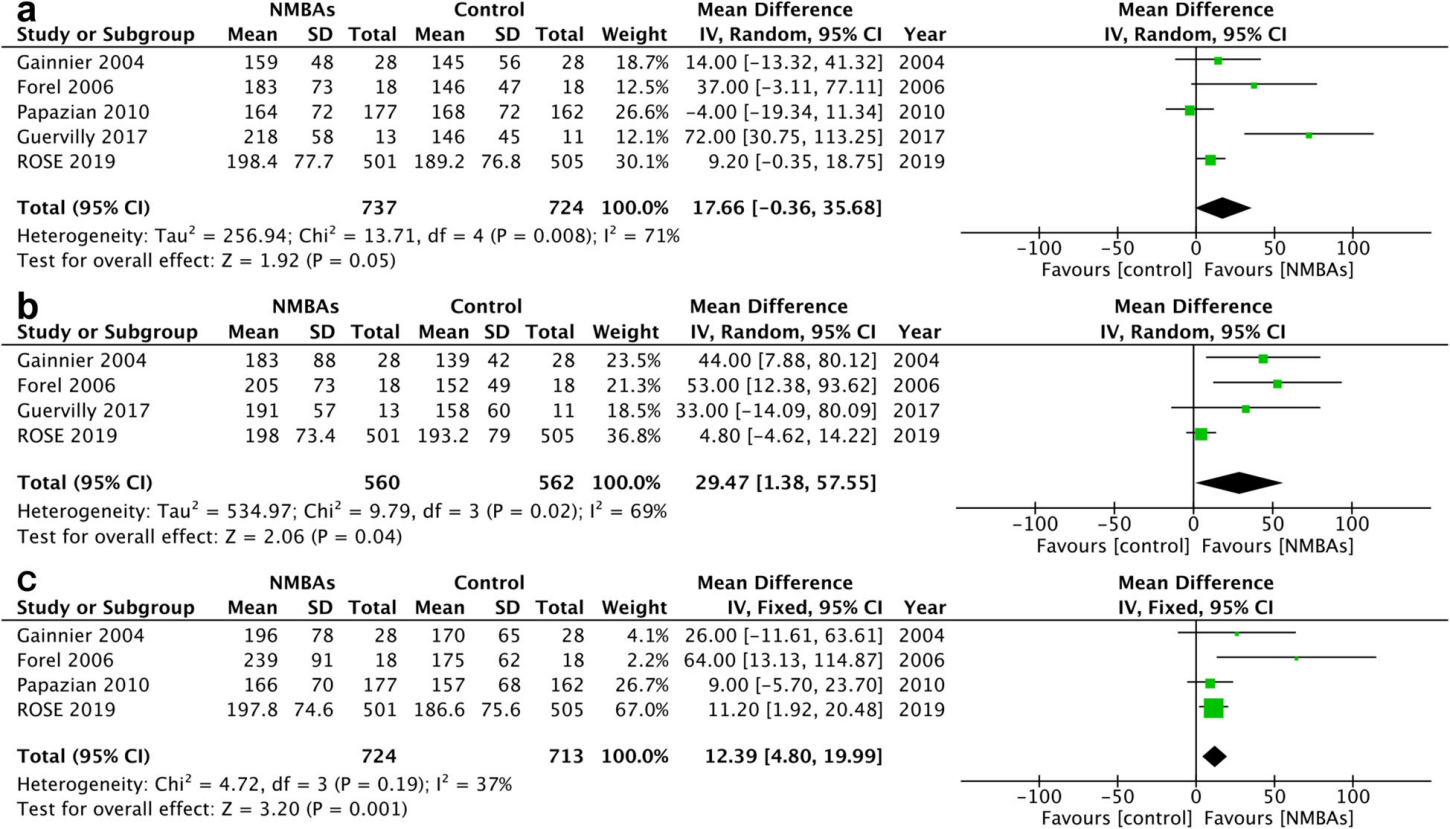
Forest plot of PaO_2_/FIO_2_. (**a**, 24 h, random effect model; **b**, 48 h, random effect model; **c**, 72 h, fixed effect model)

No statistically significant difference was found at 24 h between two groups (WMD, 17.66; 95% CI, − 0.36 to 35.68; *P* = 0.05; I^2^ = 71%; 5 trials; Fig. 3a) [9, 16–19]. The pooled analysis showed better PaO_2_/FiO_2_ in the NMBAs group at 48 h (WMD, 29.47; 95% CI, 1.38 to 57.55; *P* = 0.04; I^2^ = 69%; 4 trials; Fig. 3b) [16–19] with significant heterogeneity, and 72 h (WMD, 12.39; 95% CI, 4.80 to 19.99; *P* = 0.001; I^2^ = 37%; 4 trials; Fig. 3c) with no significant heterogeneity [9, 16–18]. Lyu [20] reported the results of PaO_2_/FiO_2_ at 48 h separately in patients with moderate and severe ARDS. We contacted the corresponding author for additional data, but received no reply. Therefore, we performed a sensitivity analysis by including moderate or severe ARDS or both, separately. The results showed better PaO_2_/FiO_2_ in the NMBAs group at 48 h when included severe ARDS alone, or both moderate and severe ARDS. No statistically significant was found if only moderate ARDS was included alone (Table 2).

##### Plateau pressure (Pplat)

**Fig. 4:**
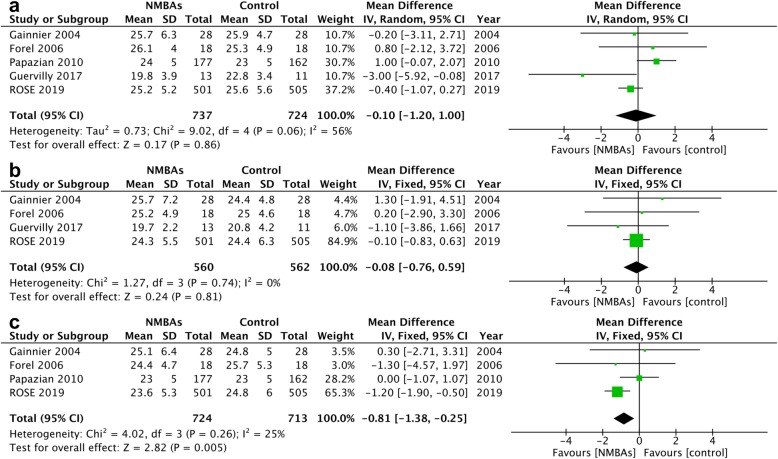
Forest plot of Plateau pressure. (**a**, 24 h, random effect model; **b**, 48 h, fixed effect model; **c**, 72 h, fixed effect model)

There was no statistical significant effects of NMBAs on Pplat at 24 h (WMD, − 0.10; 95% CI, − 1.20 to 1.00; *P* = 0.86; I^2^ = 56%; 5 trials; Fig. 4a) [9, 16–19] and 48 h (WMD, − 0.08; 95% CI, − 0.76 to 0.59; *P* = 0.81; I^2^ = 0%; 4 trials; Fig. 4b) [16–19]. Sensitivity analysis of Pplat at 48 h showed similar results by performed with random effect model (Table 2). NMBAs use could decrease the Pplat (WMD, − 0.81; 95% CI, − 1.38 to − 0.25; *P* = 0.005; I^2^ = 25%; 4 trials; Fig. 4c) at 72 h [9, 16–18], but this difference did not achieve statistical significance in random effect model (Table 2).

##### Positive end-expiratory pressure (PEEP)

**Fig. 5:**
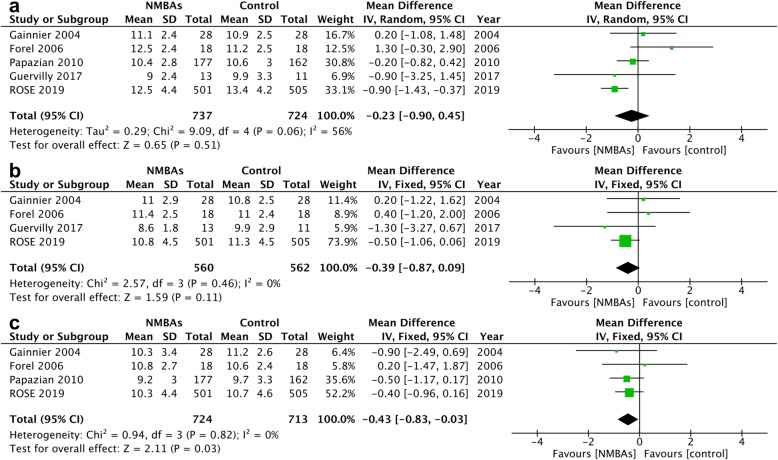
Forest plot of PEEP. (**a**, 24 h, random effect model; **b**, 48 h, fixed effect model; **c**, 72 h, fixed effect model)

The difference in PEEP between two groups did not achieve statistical significance at 24 h (WMD, − 0.23; 95% CI, − 0.90 to 0.45; *P* = 0.51; I^2^ = 56%; 5 trials; Fig. 5a) [9, 16–19] and 48 h (WMD, − 0.39; 95% CI, − 0.87 to 0.09; *P* = 0.11; I^2^ = 0%; 4 trials; Fig. 5b) [16–19]. But this difference was statistically significant (WMD, − 0.43; 95% CI, − 0.83 to − 0.03; *P* = 0.03; I^2^ = 0%; 4 trials; Fig. 5c) at 72 h [9, 16–18]. Sensitive analysis by changing the model showed similar results (Table 2).

##### Days free of ventilation at day 28 (DFV)

Five studies [9, 16–19] reported days free of ventilation at day 28. There was no difference of the DFV at day 28 (WMD, 0.55; 95% CI, − 0.46 to 1.57; *P* = 0.29; I^2^ = 13%; Additional file 1: Figure S8), either in fixed or random effect model (Table 2).

##### Days not in ICU at day 28

Three studies [9, 16, 19] reported days not in ICU at day 28. There was no statistically significant effects of NMBAs on days not in ICU at day 28 (WMD, 0.12; 95% CI, − 0.85 to 1.08; *P* = 0.82; I^2^ = 13%; Additional file 1: Figure S9), either using fixed or random effect model (Table 2).

##### Barotrauma

Five studies [9, 16–19] reported the incidence of barotrauma. NMBAs use could reduce the risk of barotrauma (RR, 0.55; 95% CI, 0.35 to 0.85; *P* = 0.007; I^2^ = 0%; Additional file 1: Figure S10) with no significant heterogeneity. Fixed and random effect model showed similar results (Table 2).

##### Effect of NMBAs on ICU-acquired weakness

Four studies [9, 16–18] reported the incidence of ICU-acquired weakness. The diagnosis of ICU-acquired weakness was made by Medical Research Council (MRC) scale in two trials [9, 16], and was not specially defined in two studies [17, 18]. We did not found that NMBAs use was associated with increased risk of ICU-acquired weakness (RR, 1.23; 95% CI, 0.99 to 1.53; *P* = 0.06; I^2^ = 0%; Additional file 1: Figure S11) with no significant heterogeneity. Detailed information about four trials reported ICU-acquired weakness were given in the Additional file 1: Table S6. Post hoc sensitive analyses showed similar results (Table 2).

##### Evidence level

A summary of the evidence level according the GRADE was presented in Additional file 1: Figure S12.

## Discussion

Our meta-analysis found that use of NMBAs in patients with ARDS might have benefits on short-term mortality, but had no significant effect on mid-term and long-term mortality. In addition, we found use of NMBAs could improve the PaO_2_/FiO_2_ ratio at 48 and 72 h, reduce the Pplat and PEEP at 72 h and was associated with less risk of barotrauma. Finally, our results showed use of NMBAs did not affect the days free of ventilation, the days not in ICU at day 28 and the risk of ICU- acquired weakness.

NMBAs may have beneficial effects on patients with ARDS through a variety of mechanisms. Such as decrease the oxygen consumption of respiratory and other muscles, reducing cardiac output, increasing the mixed venous partial pressure of oxygen, and increasing the partial pressure of arterial oxygen. By paralyzing respiratory muscles, neuromuscular blocking agents may indirectly minimize various manifestations of ventilator-induced lung injury [23]. The most used NMBAs in patients with ARDS is cisatracurium. Comparison with cisatracurium, vecuronium has different pharmacological properties [24]. Lyu [20] evaluated the effects of vecuronium in patients with moderate or severe ARDS and they found vecuronium is associated with better prognosis. However, low methodological quality may bias their results, and they did not compare the different effects between cisatracurium and vecuronium. Sottile et al. [25] compared the effects of cisatracurium with vecuronium in patients with or at risk for ARDS. They found there was no difference of mortality and hospital length of stay between two groups. Nevertheless, patients in the cisatracurium group experienced a shorter duration of mechanical ventilation and ICU length of stay. This may be because the metabolism and elimination of cisatracurium is independent of organ function and vecuronium is associated with a higher risk of ICU-acquired weakness [8]. In a United States national survey, 94% of respondents used either bolus or infusion neuromuscular blockade in patients with ARDS and 62.1% of respondents used NMBAs as tier 1 rescue strategy [26]. Due to limited evidence, recent guidelines only suggested use of NMBAs in patients with a PaO_2_/FiO_2_ less than 150 with weak recommendation [15, 27].

Three meta-analysis published separately in 2012, 2013 and 2018 reported NMBAs were associated with improved oxygenation and a lower risk of mortality and barotrauma [12, 21, 28]. However, these pooled results were affected mainly by the ACURASYS trial [9]. Our meta-analysis updated the results with the latest ROSE trial [16], which included more patients than all previous published studies. However, our results are different from the results of the previous meta-analysis. There are several possible explanations for this result. The most important factor may be the difference in sedation levels. Patients in the control group received light sedation according to the current guidelines [10, 11, 29, 30], in the ROSE trial, however, those patients received deep sedation in other previous published studies. It has been reported that deep sedation use in critically ill patients is independently associated with delayed extubation and increased mortality [31]. Moreover, Akoumianaki et al. [32] proposed a new mechanism for ventilator dyssynchrony in patients with ARDS in 2013, called reverse triggering. And deep sedation level may increase the incidence of reverse triggering, and the latter is associated with poor prognosis in patients with ARDS [33]. In addition, different median time from ARDS diagnosis to randomization, percentage of prone positioning, higher PEEP and other treatment strategies also need to be taken into consideration.

Our study showed that patients in the NMBAs group had a significant higher PaO2/FiO2 at 48 h and 72 h, and reduced Pplat and PEEP at 72 h. Although the difference of these parameters did not reach statistical significance before 48 h, but the trend toward improved results in patients who received NMBAs from 48 h to 72 h is clearly. These results indicate NMBAs therapy attenuate early hypoxemia in adult patients with ARDS. Additionally, NMBAs related complications has also been a focus of concern, especially the ICU-obtained weakness [15]. In the present study, we did not find short-term use of NMBAs could increase the incidence of ICU-obtained weakness. However, the diagnosis of ICU-obtained weakness is inconsistent and subjective and many other factors can affect the incidence of ICU-acquired weakness [34, 35]. Besides, the ROSE trial found use of cisatracurium is associated with a higher risk of serious cardiovascular events [16]. The authors speculated this may be associated with the use of deep sedation [16]. In addition, accumulation of laudanosine may increase the incidence of bradycardia and hypotension [36].

### Study strengths and limitations

The strength of our study is that the newest published multiple center RCT was included. Several limitations of our meta-analysis should be concerned. Although there was no significantly statistic heterogeneity between studies in most analytic models, it is important to note the unneglectable clinical heterogeneity. Different ARDS definition criteria, type of neuromuscular blocking agents, dosage regimens, mechanical ventilation strategies, and various adjunct treatments may bias the results. In addition, only six studies were eligible, and we cannot perform subgroup analysis according different important variables. Furthermore, the small sample sizes of four studies make our results are mainly depend on the ACURASYS [9] trial and ROSE trial [16].

### Implications for clinical practice and further researches

In the present study, we found NMBAs use is beneficial for reverse of hypoxemia and may be associated with decreased shorter-term mortality (21~28 days mortality). But they had no significantly statistical effects on long-term mortality. Along with the higher risk of serious cardiovascular events among patients received NMBAs, we do not suggest routinely use of NMBAs in all patients with ARDS. Severe ARDS patients with patient–ventilator dyssynchronies, or who are vulnerable to ventilator-induced injury may benefit from NMBAs use. Thus, we think NMBAs could be used for improvement of oxygenation in patients with severe ARDS. Additionally, further studies should focus on the following major topics. Firstly, in recent years, different subgroups or phenotypes of ARDS have been pay attention to [37]. Patients who are response to NMBAs therapy should be identified in further studies. Then, the optimal dose, time and duration of NMBAs remain unclear. The ACURASYS [9] and ROSE [16] studies used high dosages of cisatracurium (37.5 mg/h). Clinical assessment should be used in combination with Train-of-four (TOF) to titrate the optimal NMBAs’ dose [15]. The median time from ARDS diagnosis to NMBAs use is different between the ACURASYS trial [9] and ROSE trial [16] (16 h vs 7.6 h). So far, there have been no studies focusing on effects of different initiation time. Although all prospective studies limit NMBAs use within 48 h of ARDS set, there is significant heterogeneity between centers in clinical practices.

## Conclusions

In patients with respiratory distress syndrome, NMBAs may be beneficial in reverse refractory hypoxemia and may be associated with reduced short-term mortality and incidence of barotrauma. However, there is no significant effects of NMBAs on moderate and long-term mortality, and further studies are required according to insufficient evidence based on current research.

## Supplementary information

**Additional file 1: Table S1.** Study search strategy (from the inception to June 30, 2019). **Table S2.** Baseline characteristics of included trials. **Table S3.** Baseline respiratory parameters of included trials (mean ± SD). **Table S4.** Primary cause of ARDS, n (%). **Table S5.** Methodologic quality of included trials. **Table S6.** Effect of NMBAs on ICU-acquired weakness. **Figure S1.** PRISMA checklist. **Figure S2.** Risk of bias summary (each risk of bias item for each included study). **Figure S3.** Risk of bias graph (each risk of bias item presented as percentages across all included studies). **Figure S4.** Forest plot for the mortality of 28 days estimated with random effect model. **Figure S5.** Trial sequential analysis of the NMBAs on 28 days mortality. **Figure S6.** Trial sequential analysis of the NMBAs on ICU mortality. **Figure S7.** Trial sequential analysis of the NMBAs on 90 days mortality. **Figure S8.** Forest plot of DFV at day 28 estimated with fixed effect model. **Figure S9.** Forest plot of days not in ICU at day 28 estimated with fixed effect model. **Figure S10.** Forest plot of barotrauma estimated with fixed effect model. **Figure S11.** Forest plot of ICU-acquired weakness estimated with fixed effect model. **Figure S12.** GRADE summary of findings.

## Data Availability

All data generated or analyzed during the present study are included in this published article and its supplementary information files.

## Abbreviations

ARDS: acute respiratory distress syndrome
CI: confidence interval
cm: centimeter
d: day
DFV: days free of ventilation at day 28
FIO_2_: action of inspiration O_2_
GRADE: Grades of Recommendation, Assessment, Development, and Evaluation
h: hour
ICU: intensive care unit
IV: Inverse Variance
Kg: kilogram
mg: milligram
M-H: Mantel-Haenszel
min: minute
mL: milliliter
N: number
NA: not available
NMBAs: neuromuscular blocking agents
PaO_2_: partial pressure of oxygen
PEEP: positive end-expiratory pressure
Pplat: plateau pressure
PRISMA: Preferred Reporting Items for Systematic Reviews and Meta-Analyses
RR: relative risk
TSA: trial sequence analysis
WMD: weighted mean difference
μg: microgram
