# Supplementary material for: Neuromuscular blocking agents for acute respiratory distress syndrome: an updated meta-analysis of randomized controlled trials
Source: Respir Res. 2020 Jan 13;21:23. doi: 10.1186/s12931-020-1287-4 (PMC6958940; doi:10.1186/s12931-020-1287-4)
Supplement: Supplementary file 1 — Additional file 1: Table S1. Study search strategy (from the inception to June 30, 2019). Table S2. Baseline characteristics of included trials. Table S3. Baseline respiratory parameters of included trials (mean ± SD). Table S4. Primary cause of ARDS, n (%). Table S5. Methodologic quality of included trials. Table S6. Effect of NMBAs on ICU-acquired weakness. Figure S1. PRISMA checklist. Figure S2. Risk of bias summary (each risk of bias item for each included study). Figure S3. Risk of bias graph (each risk of bias item presented as percentages across all included studies). Figure S4. Forest plot for the mortality of 28 days estimated with random effect model. Figure S5. Trial sequential analysis of the NMBAs on 28 days mortality. Figure S6. Trial sequential analysis of the NMBAs on ICU mortality. Figure S7. Trial sequential analysis of the NMBAs on 90 days mortality. Figure S8. Forest plot of DFV at day 28 estimated with fixed effect model. Figure S9. Forest plot of days not in ICU at day 28 estimated with fixed effect model. Figure S10. Forest plot of barotrauma estimated with fixed effect model. Figure S11. Forest plot of ICU-acquired weakness estimated with fixed effect model. Figure S12. GRADE summary of findings. [file 12931_2020_1287_MOESM1_ESM.docx]

Contents Page

[Table S1. Study search strategy (from the inception to June 30, 2019) 2](#_Toc26696933)

[Table S2. Baseline characteristics of included trials 4](#_Toc26696934)

[Table S3. Baseline respiratory parameters of included trials (mean ± SD) 6](#_Toc26696935)

[Table S4. Primary cause of ARDS, n (%) 7](#_Toc26696936)

[Table S5. Methodologic quality of included trials 8](#_Toc26696937)

[Table S6. Effect of NMBAs on ICU-acquired weakness 10](#_Toc26696938)

[Figure S1. PRISMA checklist 11](#_Toc26696939)

[Figure S2. Risk of bias summary (each risk of bias item for each included study) 12](#_Toc26696940)

[Figure S3. Risk of bias graph (each risk of bias item presented as percentages across all included studies) 12](#_Toc26696941)

[Figure S4. Forest plot for the mortality of 28 days estimated with random effect model 13](#_Toc26696942)

[Figure S5. Trial sequential analysis of the NMBAs on 28 days mortality 14](#_Toc26696943)

[Figure S6. Trial sequential analysis of the NMBAs on ICU mortality 15](#_Toc26696944)

[Figure S7. Trial sequential analysis of the NMBAs on 90 days mortality 16](#_Toc26696945)

[Figure S8. Forest plot of DFV at day 28 estimated with fixed effect model 17](#_Toc26696946)

[Figure S9. Forest plot of days not in ICU at day 28 estimated with fixed effect model 18](#_Toc26696947)

[Figure S10. Forest plot of barotrauma estimated with fixed effect model 19](#_Toc26696948)

[Figure S11. Forest plot of ICU-acquired weakness estimated with fixed effect model 20](#_Toc26696949)

[Figure S12. GRADE summary of findings 21](#_Toc26696950)

Table S1. Study search strategy (from the inception to June 30, 2019)

| **Database** | **Block description** | **Search strategy** | **Number citation retrieved** |
| --- | --- | --- | --- |
| **PubMed** | **Patients** | #1 ("Respiratory Distress Syndrome, Adult"[Mesh] OR Respiratory Distress Syndrome, Adult OR Shock Lung OR Lung, Shock OR ARDS, Human OR ARDSs, Human OR Human ARDS OR Respiratory Distress Syndrome, Acute OR Acute Respiratory Distress Syndrome OR Adult Respiratory Distress Syndrome OR respiratory distress syndrome OR adult respiratory distress OR ARDS OR pulmonary distress syndrome OR RDS OR "Acute Lung Injury"[Mesh] OR Acute Lung Injury OR Acute Lung Injuries OR Lung Injury, Acute OR Lung Injuries, Acute OR Wet Lung OR Lung, Wet OR Lungs, Wet OR Wet Lungs) | **101775** |
|  | **Intervention** | #2 "Neuromuscular Blockade"[Mesh] OR Neuromuscular Blockade OR Blockade, Neuromuscular OR Neuromuscular Block OR Block, Neuromuscular OR "Neuromuscular Blocking Agents"[Mesh] OR Neuromuscular Blocking Agents OR Neuromuscular Blocking OR Agents, Neuromuscular Blocking OR neuromuscular blocking OR Blocking Agents, Neuromuscular OR Neuromuscular Blockers OR Blockers, Neuromuscular OR Cisatracurium OR Vecuronium | **33988** |
|  | **Study type** | #3 (clinical[tiab] AND trial[tiab]) OR "clinical trials as topic"[mesh] OR "clinical trial"[pt] OR random*[tiab] OR "random allocation"[mesh] OR "therapeutic use"[sh] | **5270289** |
|  | **Result** | #4 #1 AND #2 AND #3 | **311** |
| **EMBASE** | **Patients** | #1 'adult respiratory distress syndrome'/exp OR 'adult respiratory distress syndrome' OR 'respiratory distress syndrome, adult' OR 'shock lung' OR 'lung, shock' OR 'ards, human' OR 'ardss, human' OR 'human ards' OR 'respiratory distress syndrome, acute' OR 'acute respiratory distress syndrome' OR 'adult respiratory distress syndrome' OR 'respiratory distress syndrome' OR 'adult respiratory distress' OR 'ards' OR 'pulmonary distress syndrome' OR 'rds' OR 'acute lung injury'/exp OR 'acute lung injury' OR "acute lung injuries' OR 'lung injury, acute' OR 'lung injuries, acute' OR 'wet lung' OR 'lung, wet' OR 'lungs, wet' OR 'wet lungs' | **86921** |
|  | **Intervention** | #2 'neuromuscular blocking'/exp OR 'neuromuscular blocking' OR 'neuromuscular blockade' OR 'blockade, neuromuscular' OR 'neuromuscular block' OR 'block, neuromuscular' OR 'neuromuscular blocking agent'/exp OR 'neuromuscular blocking agent' OR 'agents, neuromuscular blocking' OR 'blocking agents, neuromuscular' OR 'neuromuscular blockers' OR 'blockers, neuromuscular' OR 'cisatracurium' OR 'vecuronium' | **82025** |
|  | **Study type** | #3 ('clinical':ti,ab AND 'trial':ti,ab) OR 'clinical trial'/exp OR random* OR 'drug therapy':lnk | **5482136** |
|  | **Result** | 4 #1 AND #2 AND #3 | **662** |
| **Cochrane library (Cochrane Central Register of Controlled Trials)** | **Patients** | #1 MeSH descriptor: [Respiratory Distress Syndrome, Adult] explode all trees  #2 MeSH descriptor: [Acute Lung Injury] explode all trees  #3 Respiratory Distress Syndrome, Adult OR Shock Lung OR Lung, Shock OR ARDS, Human OR ARDSs, Human OR Human ARDS OR Respiratory Distress Syndrome, Acute OR Acute Respiratory Distress Syndrome OR Adult Respiratory Distress Syndrome OR respiratory distress syndrome OR adult respiratory distress OR ARDS OR pulmonary distress syndrome OR RDS OR Acute Lung Injury OR Acute Lung Injuries OR Lung Injury, Acute OR Lung Injuries, Acute OR Wet Lung OR Lung, Wet OR Lungs, Wet OR Wet Lungs  #4 #1 OR #2 OR #3 | **8384** |
|  | **Intervention** | #5 MeSH descriptor: [Neuromuscular Blockade] explode all trees  #6 MeSH descriptor: [Neuromuscular Blocking Agents] explode all trees  #7 Neuromuscular Blockade OR Blockade, Neuromuscular OR Neuromuscular Block OR Block, Neuromuscular OR neuromuscular blocking OR Neuromuscular Blocking Agents OR Agents, Neuromuscular Blocking OR Blocking Agents, Neuromuscular OR Neuromuscular Blockers OR Blockers, Neuromuscular OR Cisatracurium OR Vecuronium  #8 #5 #6 OR #7 | **5492** |
|  | **Result** | #4 AND #8 | **94** |

Table S2. Baseline characteristics of included trials

| Study | Study period | Length of enrollment | Main Outcomes Evaluated | NMBAs used in control group (n, %) | Total mortality (n, %) |
| --- | --- | --- | --- | --- | --- |
| Gainnier  2004[1] | September 1, 2000 to December 1, 2001 | Enrolled within 36 hours when the eligibility criteria were first met | oxygenation, PEEP, Pplat, ventilator-free days, ICU mortality, barotrauma, clinically suspected critical illness neuromyopathy | 2 (7.1) | 35 (62.5) |
| Forel  2006[2] | June 2002 to December 2003 | After randomization, patients received either conventional therapy plus NMBAs (NMBA group) or conventional therapy plus placebo (control group) for the next 48 hours | Inflammatory cytokines of pulmonary and systemic inflammatory response, oxygenation | 0 (0) | 15 (41.7) |
| Papazian  2010[3] | March 2006 to March 2008 | The median time from the diagnosis of ARDS to study inclusion was 16 hours (interquartile range, 6 to 29) | 90-day mortality, 28-day mortality, days outside the ICU, days without organ or system failure, barotrauma, ICU-acquired paresis, ventilator-free days | 36 (22.0) | 124 (36.6) |
| Lyu  2014[4] | July 2012 to September 2013 | NA | 21-day mortality, oxygenation, APACHE II score, SOFA score, lactate, C-reactive protein | NA | 27 (28.1) |
| Guervilly  2017[5] | NA | NA | transpulmonary pressures, severity scores, oxygenation, Pplat, days outside the ICU, ventilator-free days, 28-day mortality, ICU mortality | NA | 8 (33.3) |
| ROSE  2019[6] | January 2016 to April 2018 | Patients were enrolled a median of 7.6 hours (interquartile range, 3.7 to 15.6) after diagnosis of moderate-to-severe ARDS | 90-day mortality, organ dysfunction, 28-day mortality, days free of organ dysfunction, days not in the ICU, ventilator-free days, days not in the hospital at day 28 | 86 (17.0) | 429 (42.6) |
| NA, not available; PEEP, positive end-expiratory pressure; Pplat, plateau pressure; ICU, intensive care unit; NMBAs, neuromuscular blocking agents; ARDS, Acute respiratory distress syndrome; APACHE II, acute physiology and chronic health evaluation II; SOFA, sequential organ failure assessment | | | | | |

Table S3. Baseline respiratory parameters of included trials (mean ± SD)

|  | Gainnier 2004[1] | | Forel 2006[2] | | Papazian 2010[3] | | Lyu 2014[4] | | | | Guervilly 2017[5] | | ROSE 2019[6] | |
| --- | --- | --- | --- | --- | --- | --- | --- | --- | --- | --- | --- | --- | --- | --- |
|  | **NMBAs** | **Control** | **NMBAs** | **Control** | **NMBAs** | **Control** | **NMBAs (moderate)** | **Control (moderate)** | **NMBAs (severe)** | **Control (severe)** | **NMBAs** | **Control** | **NMBAs** | **Control** |
| PaO_2_/FIO_2_ | 130 ± 34 | 119 ± 31 | NA | NA | 106 ± 36 | 115 ± 41 | 140.95 ± 26.97 | 144.33 ± 24.09 | 77.68 ± 11.21 | 80.61 ± 12.82 | 158.0 ± 44.9* | 152.7 ± 56.0* | 116.1 ± 38.3 | 115.8 ± 40.1 |
| Pplat | 27.1 ± 6.2 | 26.1 ± 4.0 | 27.5 ± 4.4 | 24.8 ± 5.7 | 25.0 ± 5.1 | 24.4 ± 4.7 | NA | NA | NA | NA | 22.7 ± 5.8* | 21.7 ± 5.1* | 25.5 ± 6.0 | 25.7 ± 6.1 |
| PEEP | 12.3 ± 3.0 | 11.4 ± 2.5 | 13.2 ± 2.7 | 11.0 ± 2.7 | 9.2 ± 3.2 | 9.2 ± 3.5 | NA | NA | NA | NA | 10.8 ± 1.2* | 10.3 ± 2.5* | 12.6 ± 3.6 | 12.5 ± 3.6 |
| NA: Not available; NMBAs, neuromuscular blocking agents; FIO2, action of inspiration O2; PaO2, partial pressure of oxygen; Pplat, plateau pressure; *, the result is calculated from the median and interquartile range | | | | | | | | | | | | | | |

Table S4. Primary cause of ARDS, n (%)

|  | Gainnier 2004[1] | Forel 2006[2] | Papazian 2010[3] | Lyu 2014[4] | Guervilly 2017[5] | ROSE 2019[6] |
| --- | --- | --- | --- | --- | --- | --- |
| Pneumonia for all causes | 27(48.2) | 28 (77.8) | 201 (59.3) | 36 (37.5) | 11 (45.8) | 593 (58.9) |
| Aspiration pneumonia for all causes | 14 (25.0) | 0 (0.0) | 64 (18.9) | 0 (0.0) | 10 (41.7) | 166 (16.5) |
| Extrapulmonary sepsis for all causes | 0 (0.0) | 0 (0.0) | 40 (11.8) | 60 (62.5) | 3 (12.5) | 139 (13.8) |
| Septic shock | 6 (10.7) | 0 (0.0) | 16 (4.7) | 0 (0.0) | 20 (83.3) | 0 (0.0) |
| Trauma/Lung contusion | 3 (5.4) | 1 (2.8) | 8 (2.4) | 0 (0.0) | 0 (0.0) | 39 (3.9) |
| Others | 6 (10.7) | 0 (0.0) | 28 (8.3) | 0 (0.0) | 0 (0.0) | 108 (10.7) |
| # Repeat counts may exist due to miscellaneous diseases | | | | | | |

Table S5. Methodologic quality of included trials

| Study | Random sequence generation (selection bias) | Allocation  Concealment  (selection bias) | Blinding of participants and personnel  (performance bias) | Blinding of outcome  assessment (detection bias) | Incomplete outcome data (attrition bias) | Selective reporting (reporting bias) | Other bias |
| --- | --- | --- | --- | --- | --- | --- | --- |
| Gainnier  2004[1] | **Low risk** | **Low risk** | **High risk** | **Unclear risk** | **Low risk** | **Low risk** | **Low risk** |
|  | Computer-generated random number  sequences | Centralized | Nonblinded nurses,  a sheet masked the  infusion pump | Not reported | Not reported | Not reported | Not reported |
| Forel  2006[2] | **Low risk** | **Low risk** | **High risk** | **Unclear risk** | **Low risk** | **Low risk** | **Low risk** |
|  | Computer-generated random number  sequences | Centralized | Nonblinded nurses | Not reported | Not reported | Not reported | Not reported |
| Papazian  2010[3] | **Low risk** | **Low risk** | **Low risk** | **Low risk** | **Low risk** | **Low risk** | **Low risk** |
|  | Computer-generated random number  sequences | Centralized,  undisclosed block sizes | Blinding of patients,  clinicians, evaluators,  investigators, analysis | Not reported | Not reported | Not reported | Not reported |
| Lyu  2014[4] | **Low risk** | **Unclear risk** | **Unclear risk** | **Unclear risk** | **Low risk** | **Low risk** | **Low risk** |
|  | Random number  table | Not reported | Not reported | Not reported | Not reported | Not reported | Not reported |
| Guervilly  2017[5] | **Low risk** | **Low risk** | **Unclear risk** | **Unclear risk** | **Low risk** | **Low risk** | **Low risk** |
|  | Computer-generated random number  table | Centralized | Not reported | Not reported | Not reported | Not reported | Not reported |
| ROSE  2019[6] | **Low risk** | **Low risk** | **High risk** | **Low risk** | **Low risk** | **Low risk** | **Low risk** |
|  | Coordinating center web based  randomization system | Centralized, a computer-generated study ID  number and study arm  assignment to  neuromuscular blockade or control | Treatment was not  administered in a blinded  manner bed nurses,  a sheet masked the  infusion pump | Postdischarge outcomes were assessed by investigators  unaware of assignment.  Not sure about blinding of  in-hospital outcome  assessment | Not reported | Not reported | Not reported |

Table S6. Effect of NMBAs on ICU-acquired weakness

| Study | Patients (n) | NMBAs | Experimental (events/total, n) | Control (events/total, n) | Evaluation method | Evaluation period |
| --- | --- | --- | --- | --- | --- | --- |
| Gainnier  2004[1] | 56 | cisatracurium | 0/28 | 0/18 | clinically detectable critical illness neuromyopathy | Not avaliable |
| Forel  2006[2] | 36 | cisatracurium | 1/18 | 1/18 | clinically detectable critical illness neuromyopathy | Not avaliable |
| Papazian  2010[3] | 339 | cisatracurium | 40/177 | 28/162 | Medical Research Council (MRC) scale | the MRC scores on day 28 and at the time of ICU discharge |
| ROSE  2019[6] | 1006 | cisatracurium | 107/501 | 89/505 | Medical Research Council (MRC) scale is < 48 (or mean MRC < 4 for each muscle group tested) | Manual muscle strength testing will be attempted at study day 7, and then every 7 days thereafter, until hospital discharge or day 28 (whichever comes first) |

Figure S1. PRISMA checklist


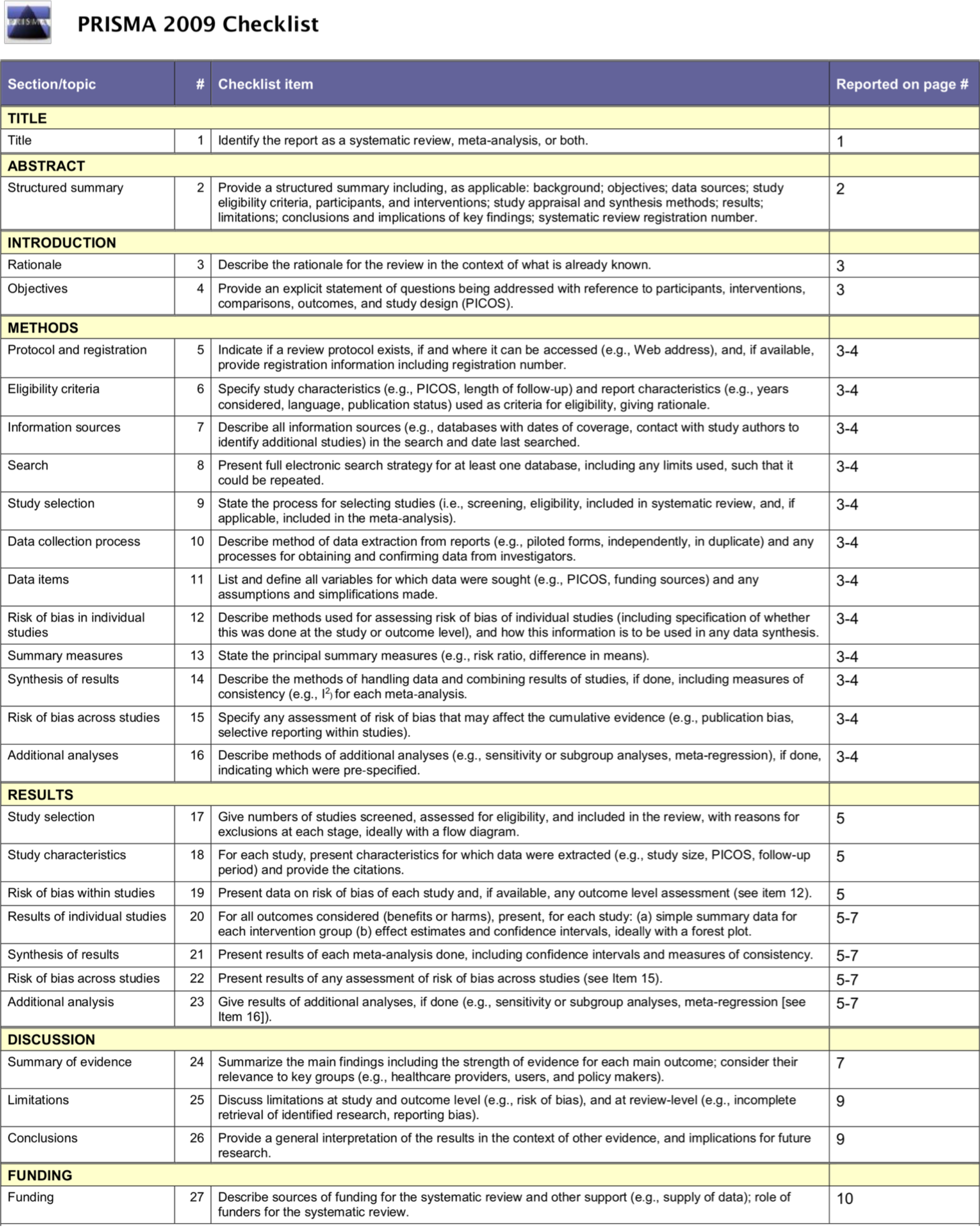


Figure S2. Risk of bias summary (each risk of bias item for each included study)

**
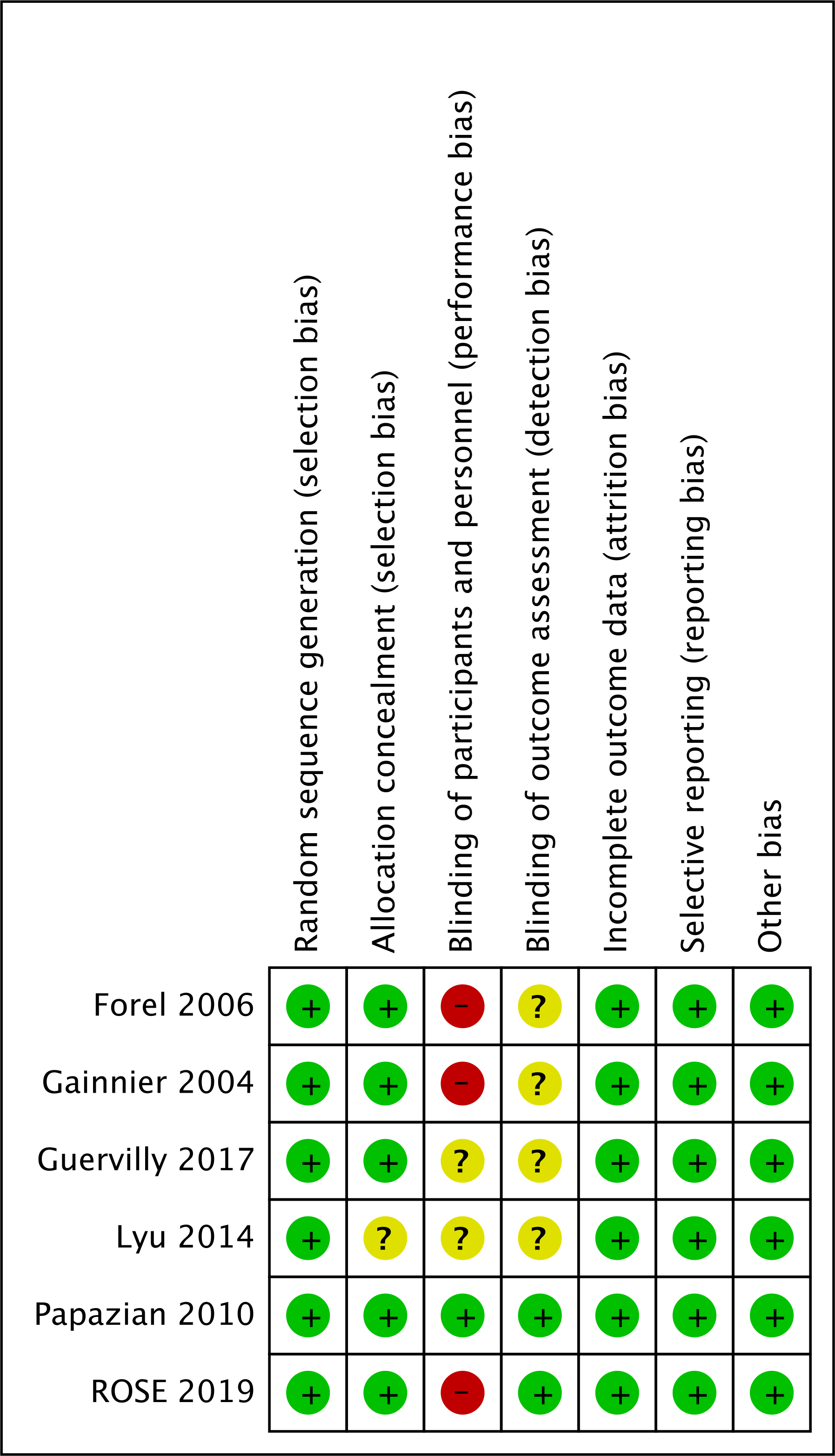
**

Figure S3. Risk of bias graph (each risk of bias item presented as percentages across all included studies)


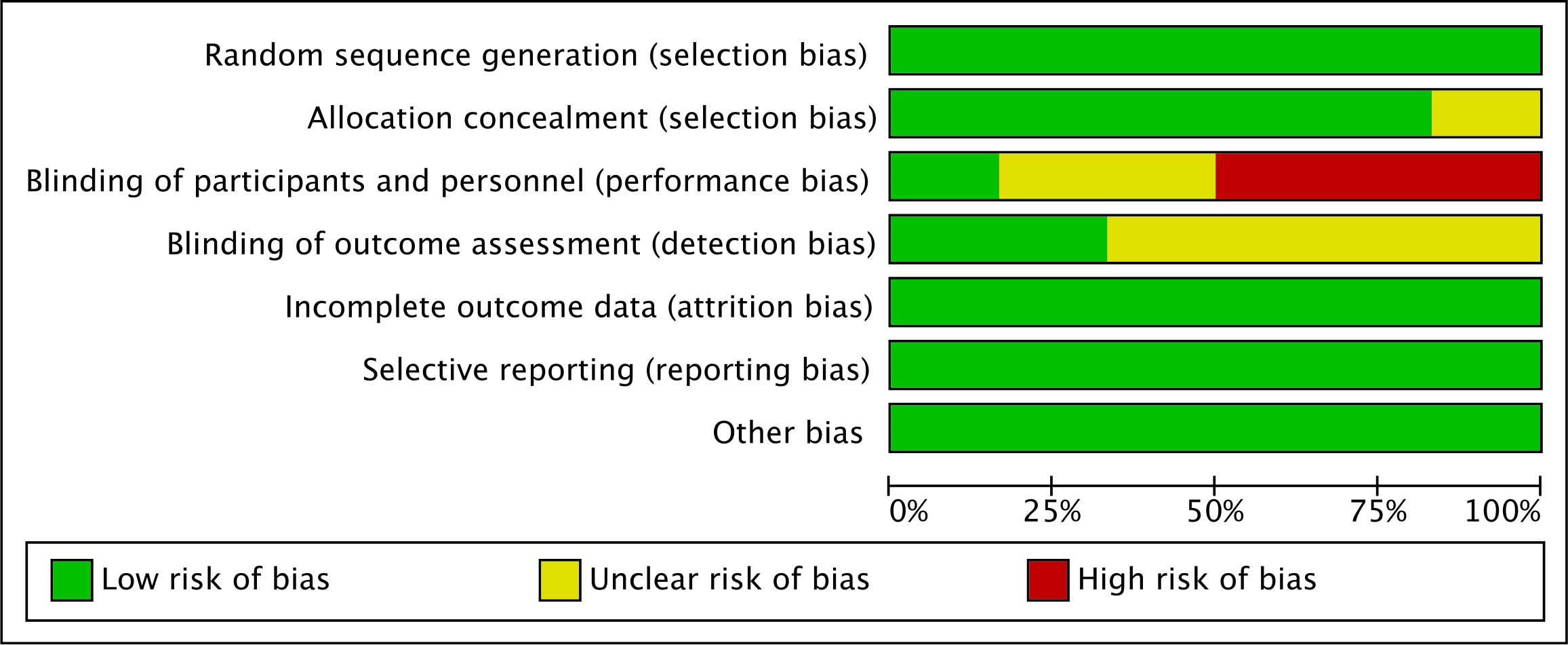


Figure S4. Forest plot for the mortality of 28 days estimated with random effect model

**
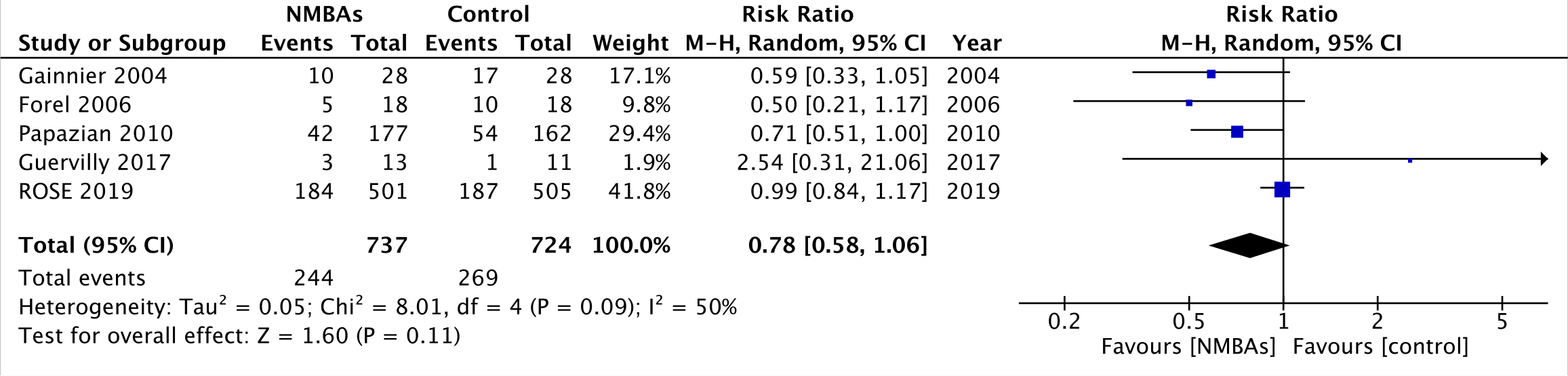
**

Figure S5. Trial sequential analysis of the NMBAs on 28 days mortality

(The optimal information size of 19729 patients was calculated using α = 0.05 (two-sided), β = 0.20 (power 95%), an anticipated relative risk reduction of 11%, and an event proportion of 37.2% in the control arm. The blue cumulative z curve was constructed using a fixed effects model.)


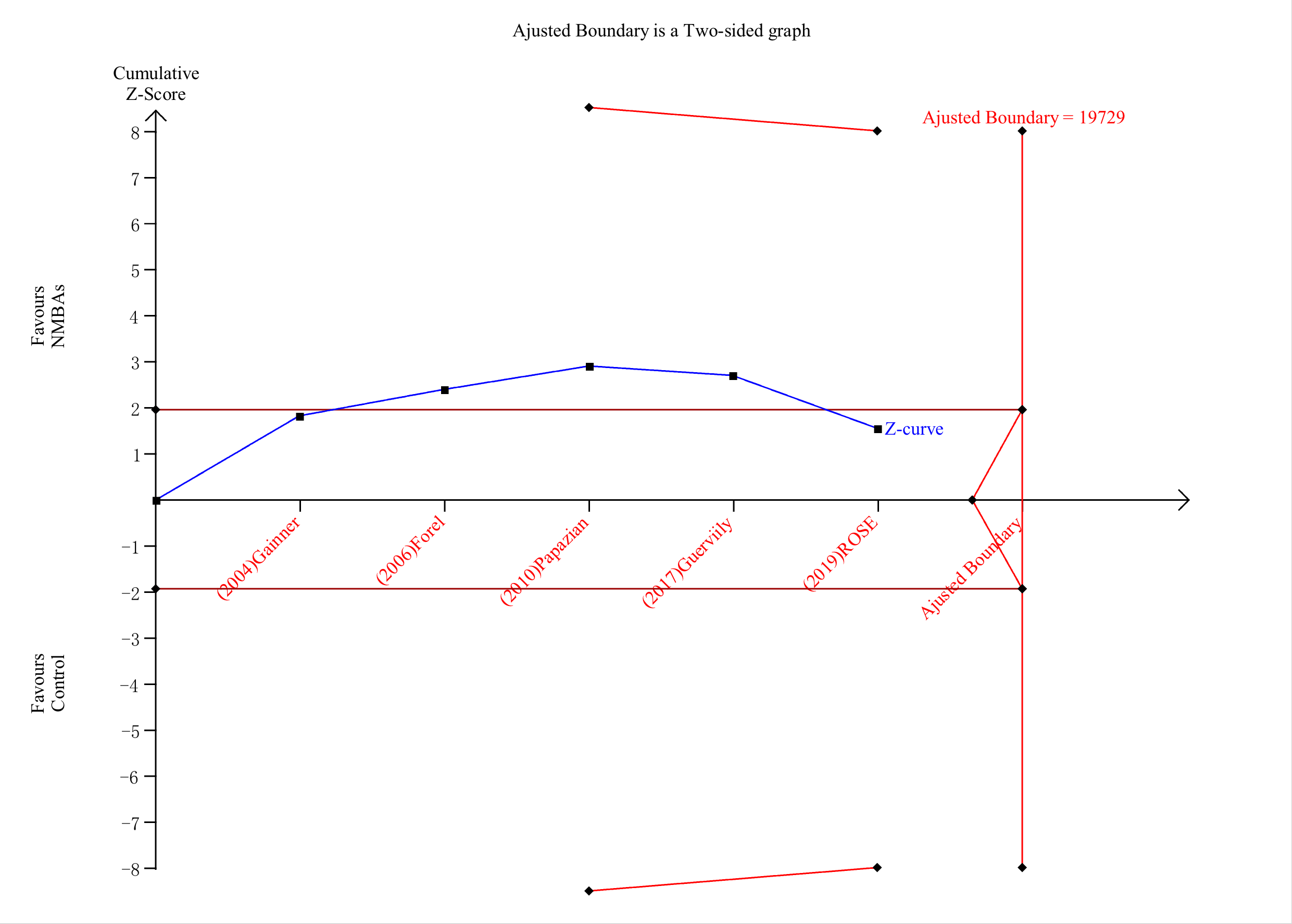


Figure S6. Trial sequential analysis of the NMBAs on ICU mortality

(The optimal information size of 21222 patients was calculated using α = 0.05 (two-sided), β = 0.20 (power 95%), an anticipated relative risk reduction of 10.2%, and an event proportion of 39.1% in the control arm. The blue cumulative z curve was constructed using a fixed effects model.)


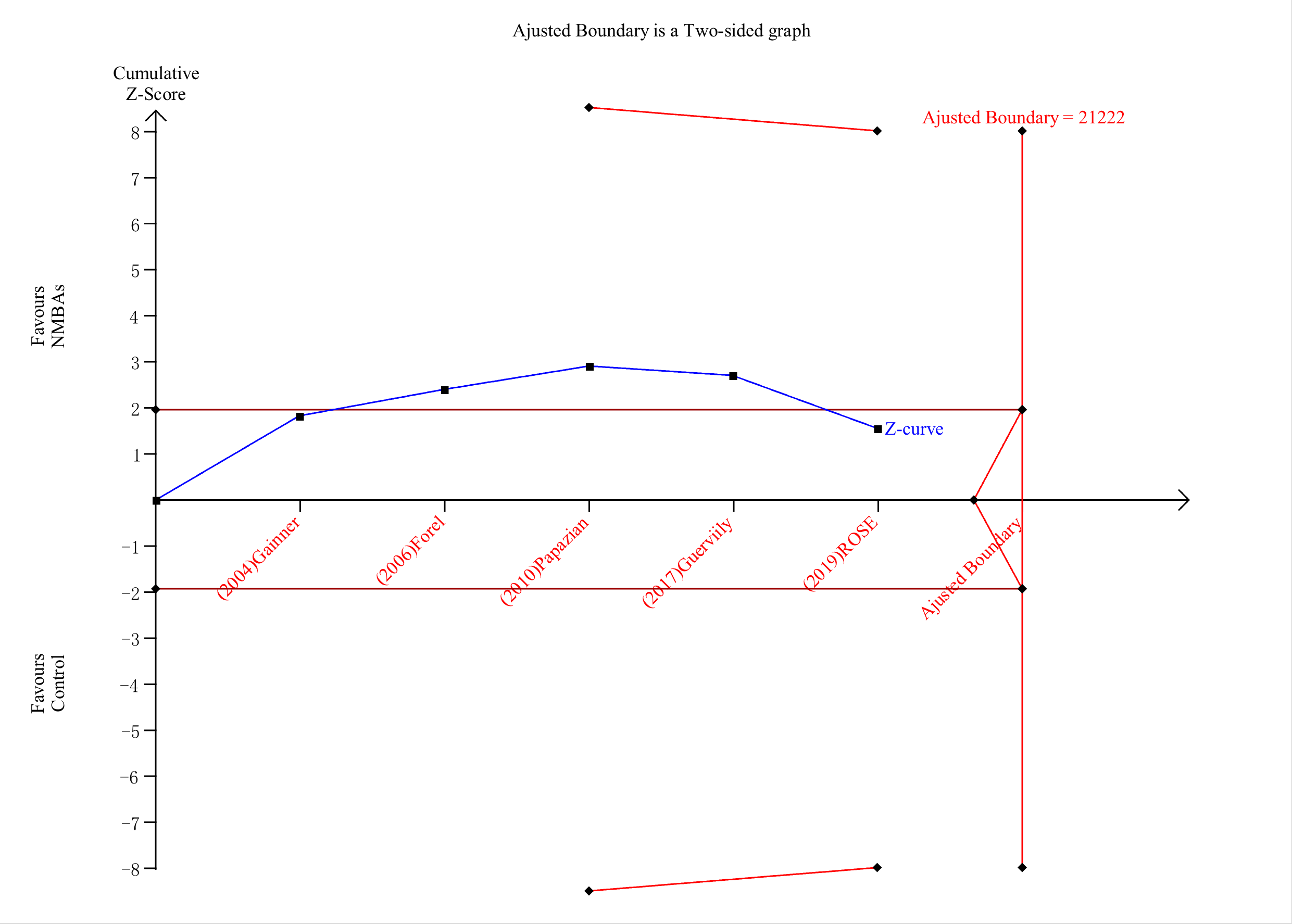


Figure S7. Trial sequential analysis of the NMBAs on 90 days mortality

(The optimal information size of 25699 patients was calculated using α = 0.05 (two-sided), β = 0.20 (power 95%), an anticipated relative risk reduction of 8.5%, and an event proportion of 43.5% in the control arm. The blue cumulative z curve was constructed using a fixed effects model.)


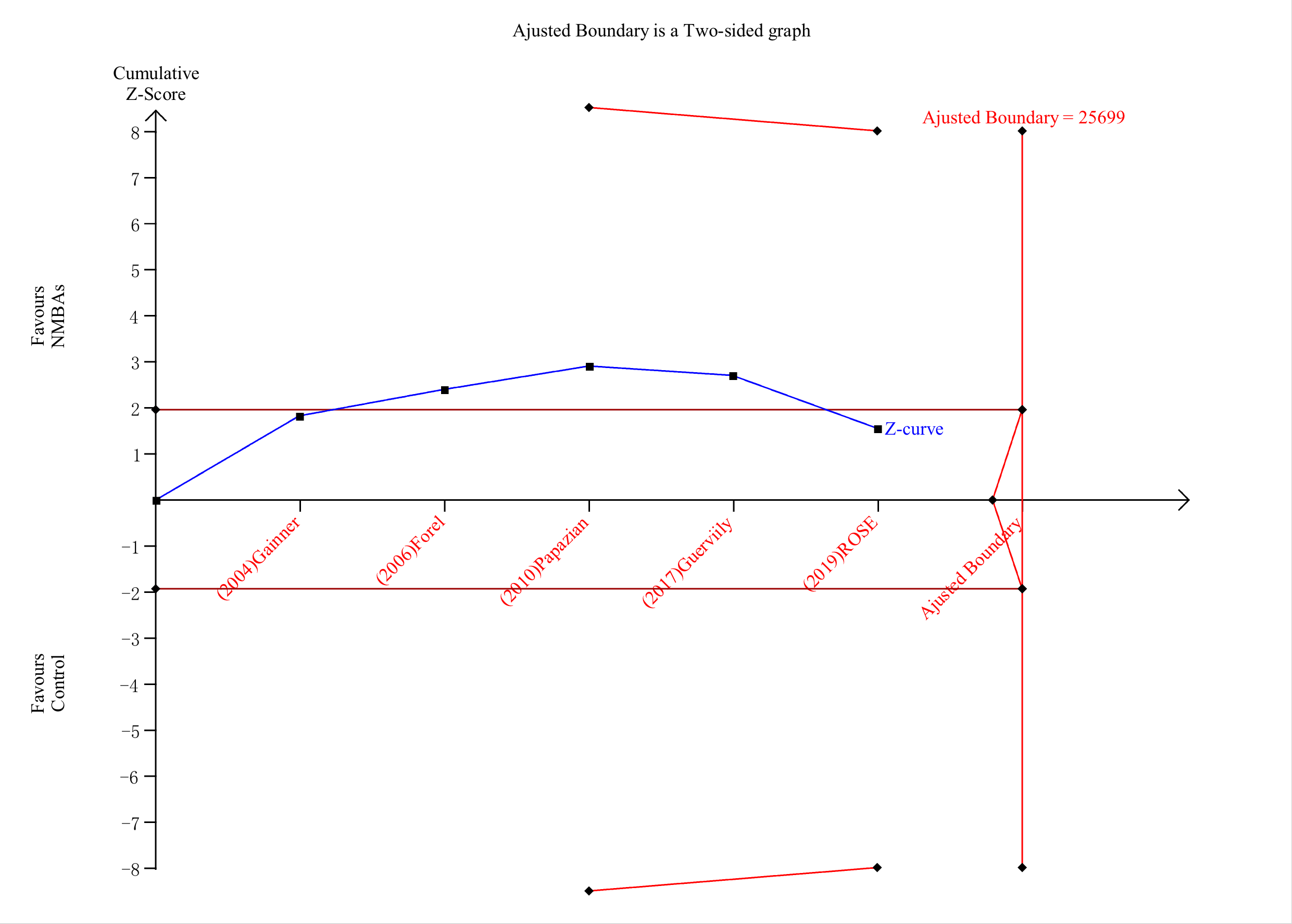


Figure S8. Forest plot of DFV at day 28 estimated with fixed effect model

**
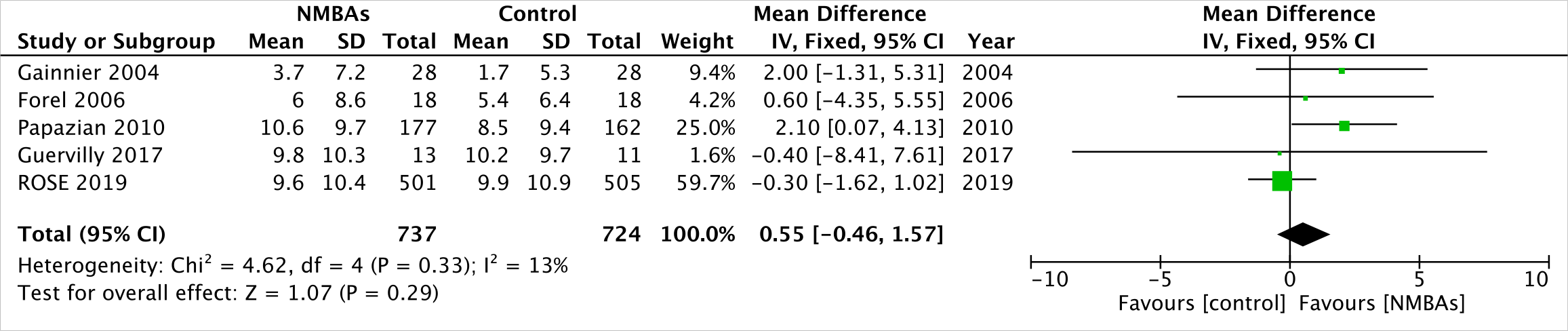
**

Figure S9. Forest plot of days not in ICU at day 28 estimated with fixed effect model

**
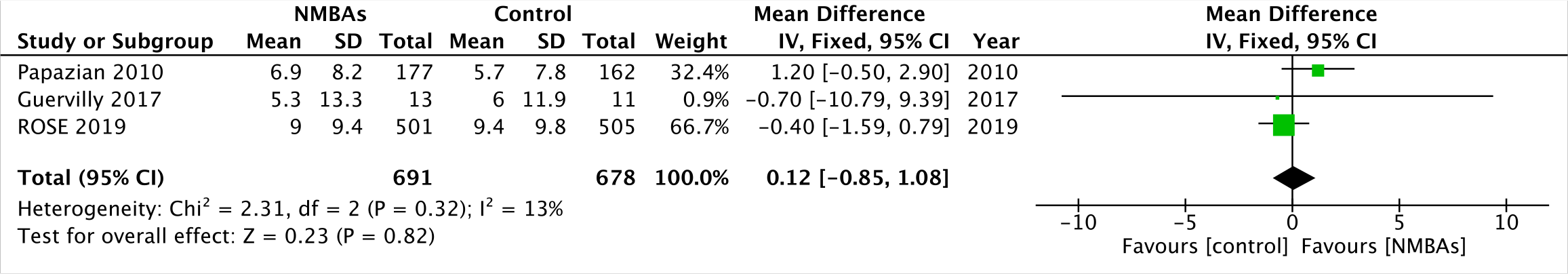
**

Figure S10. Forest plot of barotrauma estimated with fixed effect model


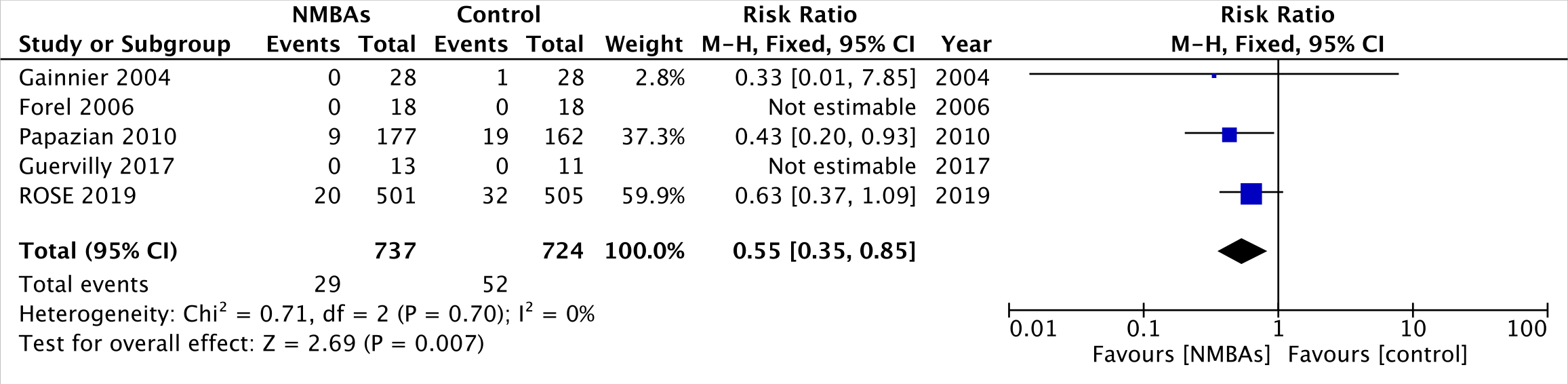


Figure S11. Forest plot of ICU-acquired weakness estimated with fixed effect model

**
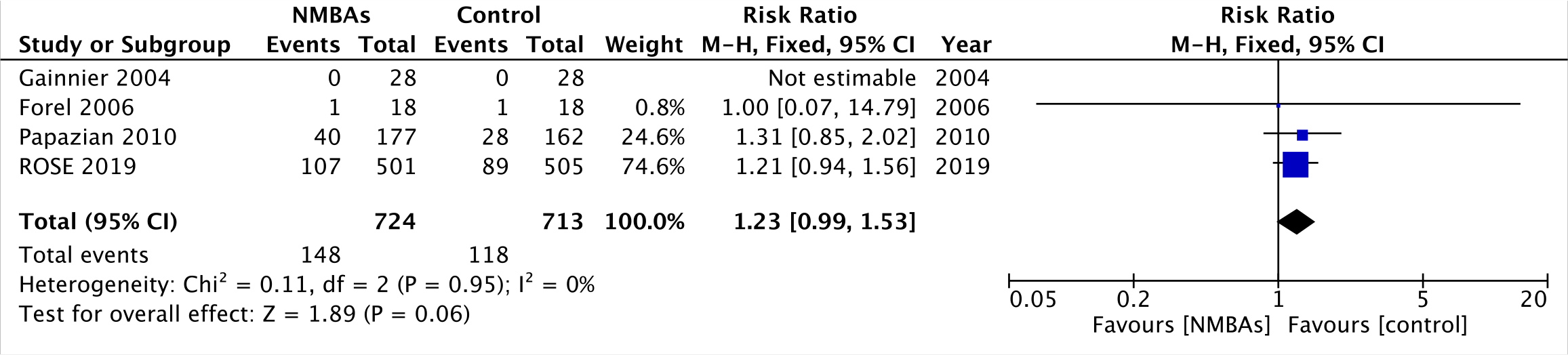
**

Figure S12. GRADE summary of findings

(Results pooled with random effect model: 21 to 28 days mortality, ICU mortality and 90 days mortality; Results pooled with fixed effect model: biotrauma, days free of ventilation at day 28; days not in ICU at day 28 and ICU-acquired weakness)

**
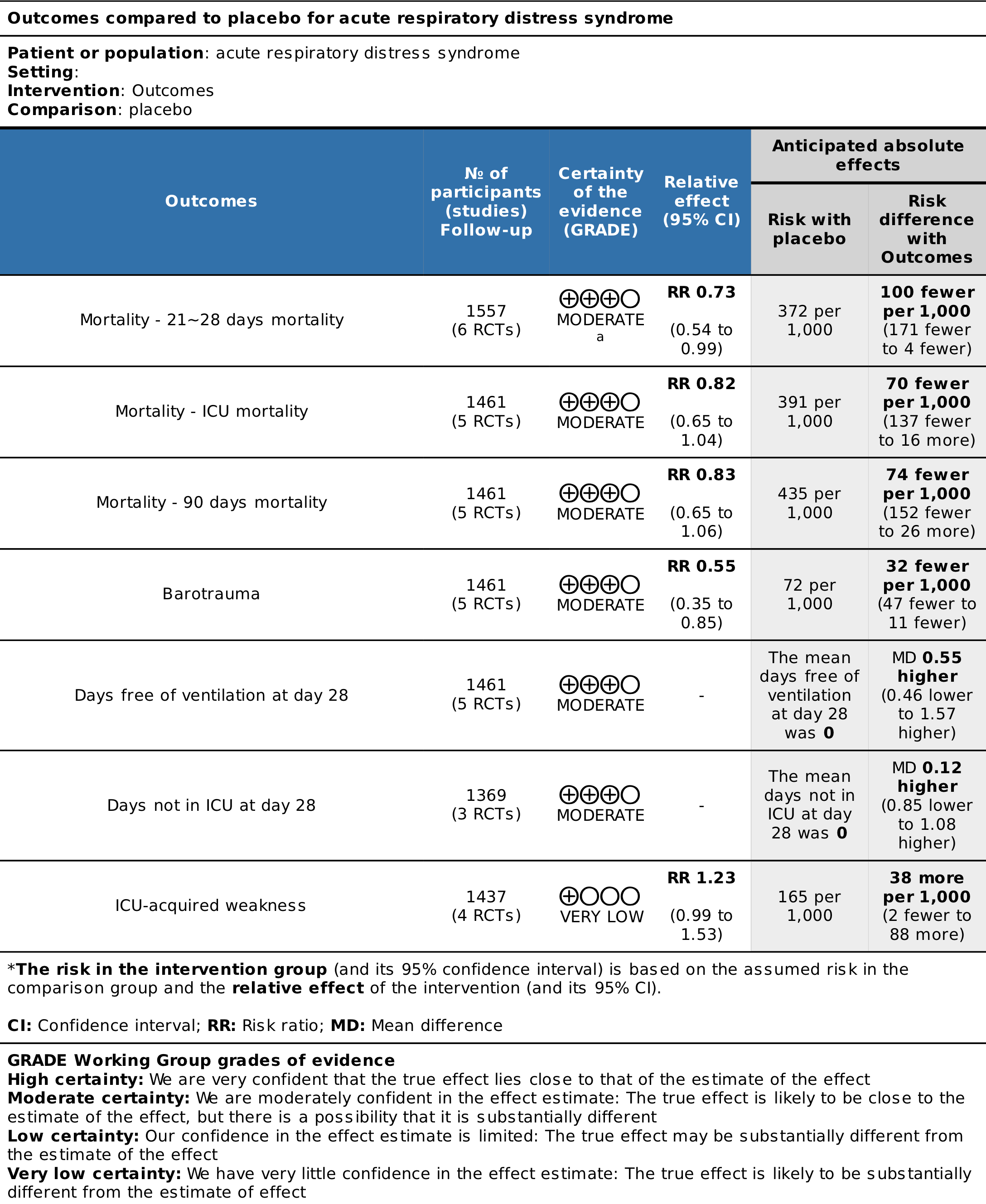
**

**References**

1. Gainnier M, Roch A, Forel JM, Thirion X, Arnal JM, Donati S, Papazian L (2004) Effect of neuromuscular blocking agents on gas exchange in patients presenting with acute respiratory distress syndrome. Crit Care Med 32 (1):113-119. doi:10.1097/01.ccm.0000104114.72614.bc

2. Forel JM, Roch A, Marin V, Michelet P, Demory D, Blache JL, Perrin G, Gainnier M, Bongrand P, Papazian L (2006) Neuromuscular blocking agents decrease inflammatory response in patients presenting with acute respiratory distress syndrome. Crit Care Med 34 (11):2749-2757. doi:10.1097/01.ccm.0000239435.87433.0d

3. Papazian L, Forel JM, Gacouin A, Penot-Ragon C, Perrin G, Loundou A, Jaber S, Arnal JM, Perez D, Seghboyan JM, Constantin JM, Courant P, Lefrant JY, Guerin C, Prat G, Morange S, Roch A (2010) Neuromuscular blockers in early acute respiratory distress syndrome. The New England journal of medicine 363 (12):1107-1116. doi:10.1056/NEJMoa1005372

4. Lyu G, Wang X, Jiang W, Cai T, Zhang Y (2014) [Clinical study of early use of neuromuscular blocking agents in patients with severe sepsis and acute respiratory distress syndrome]. Zhonghua wei zhong bing ji jiu yi xue 26 (5):325-329. doi:10.3760/cma.j.issn.2095-4352.2014.05.008

5. Guervilly C, Bisbal M, Forel JM, Mechati M, Lehingue S, Bourenne J, Perrin G, Rambaud R, Adda M, Hraiech S, Marchi E, Roch A, Gainnier M, Papazian L (2017) Effects of neuromuscular blockers on transpulmonary pressures in moderate to severe acute respiratory distress syndrome. Intensive care medicine 43 (3):408-418. doi:10.1007/s00134-016-4653-4

6. Moss M, Huang DT, Brower RG, Ferguson ND, Ginde AA, Gong MN, Grissom CK, Gundel S, Hayden D, Hite RD, Hou PC, Hough CL, Iwashyna TJ, Khan A, Liu KD, Talmor D, Thompson BT, Ulysse CA, Yealy DM, Angus DC (2019) Early Neuromuscular Blockade in the Acute Respiratory Distress Syndrome. The New England journal of medicine 380 (21):1997-2008. doi:10.1056/NEJMoa1901686
